# *In situ* Spectroscopy Reveals that Microorganisms in Different Phyla Use Different Electron Transfer Biomolecules to Respire Aerobically on Soluble Iron

**DOI:** 10.3389/fmicb.2016.01963

**Published:** 2016-12-08

**Authors:** Robert C. Blake II, Micah D. Anthony, Jordan D. Bates, Theresa Hudson, Kamilya M. Hunter, Brionna J. King, Bria L. Landry, Megan L. Lewis, Richard G. Painter

**Affiliations:** ^1^College of Pharmacy, Xavier University of Louisiana, New OrleansLA, USA; ^2^Department of Biology, Xavier University of Louisiana, New OrleansLA, USA

**Keywords:** *in situ* spectroscopy, aerobic respiration on iron, electron transport chains, cytochromes, chemolithotrophic bacteria

## Abstract

Absorbance spectra were collected on 12 different live microorganisms, representing six phyla, as they respired aerobically on soluble iron at pH 1.5. A novel integrating cavity absorption meter was employed that permitted accurate absorbance measurements in turbid suspensions that scattered light. Illumination of each microorganism yielded a characteristic spectrum of electrochemically reduced colored prosthetic groups. A total of six different patterns of reduced-minus-oxidized difference spectra were observed. Three different spectra were obtained with members of the Gram-negative eubacteria. *Acidithiobacillus*, representing Proteobacteria, yielded a spectrum in which cytochromes *a* and *c* and a blue copper protein were all prominent. *Acidihalobacter*, also representing the Proteobacteria, yielded a spectrum in which both cytochrome *b* and a long-wavelength cytochrome *a* were clearly visible. Two species of *Leptospirillum*, representing the Nitrospirae, both yielded spectra that were dominated by a cytochrome with a reduced peak at 579 nm. *Sulfobacillus* and *Alicyclobacillus*, representing the Gram-positive Firmicutes, both yielded spectra dominated by *a*-type cytochromes. *Acidimicrobium* and *Ferrimicrobium*, representing the Gram-positive Actinobacteria, also yielded spectra dominated by *a*-type cytochromes. *Acidiplasma* and *Ferroplasma*, representing the Euryarchaeota, both yielded spectra dominated by a *ba*_3_-type of cytochrome. *Metallosphaera* and *Sulfolobus*, representing the Crenarchaeota, both yielded spectra dominated by the same novel cytochrome as that observed in the Nitrospirae and a new, heretofore unrecognized redox-active prosthetic group with a reduced peak at around 485 nm. These observations are consistent with the hypothesis that individual acidophilic microorganisms that respire aerobically on iron utilize one of at least six different types of electron transfer pathways that are characterized by different redox-active prosthetic groups. *In situ* absorbance spectroscopy is shown to be a useful complement to existing means of investigating the details of energy conservation in intact microorganisms under physiological conditions.

## Introduction

The capacity to respire aerobically on soluble ferrous ions under strongly acidic conditions (pH < 3) is currently thought to be expressed in 42 species distributed among 19 genera in six phyla (Bonnefoy and Holmes, 2012; Johnson et al., 2012). It is generally accepted that the electron donor, soluble iron, does not enter the cytoplasm in appreciable quantities in any of these microorganisms. Consequently, microorganisms must express appropriate electron transfer biomolecules to conduct respiratory electrons from the extracellular iron to the intracellular molecular oxygen that serves as the terminal electron acceptor. A variety of different electron transfer proteins and redox-active prosthetic groups have been posited by many laboratories to participate in aerobic respiration on iron. These reports include studies using purified proteins and protein complexes (Cox and Boxer, 1978; Kai et al., 1992; Blake and Shute, 1997; Castelle et al., 2008; Singer et al., 2008; Bandeiras et al., 2009), spectroscopic studies on cell-free extracts (Hart et al., 1991; Blake et al., 1993; Yarzabal et al., 2002; Brasseur et al., 2004; Kappler et al., 2005; Bathe and Norris, 2007; Dinarieva et al., 2010), inventories of putative respiratory proteins deduced from whole-cell genomic sequencing activities (reviewed in Auernik and Kelly, 2008; Kozubal et al., 2011; Ilbert and Bonnefoy, 2013), and lists of likely respiratory proteins identified in transcriptomic (Auernik and Kelly, 2008; Quatrini et al., 2009) and proteomic (Dopson et al., 2005; Ram et al., 2005; Bouchal et al., 2006; Valenzuela et al., 2006) studies of cells cultured in the presence of soluble iron.

This laboratory initiated a new systems approach to study respiratory electron transfer in intact cells using a novel integrating cavity absorption meter (ICAM) that permitted the acquisition of accurate absorbance data in suspensions of cells that scattered light (Blake and Griff, 2012; Li et al., 2015). The observation chamber of this spectrophotometer comprised a reflecting cavity that was completely filled with the colored suspension of intact microorganisms. This physical arrangement permitted the suspensions of live bacteria to be irradiated in an isotropic homogeneous field of incident measuring light where the absorbed radiant power was expected to be independent of scattering effects (Elterman, 1970; Fry et al., 1992; Javorfi et al., 2006; Hodgkinson et al., 2009). When intact cells of either *Leptospirillum ferrooxidans* or *Acidithiobacillus ferrooxidans* were exposed to soluble ferrous ions under physiological solution conditions, the reduced forms of selected redox-active cellular prosthetic groups were immediately apparent in the absorbance spectrum of each organism. When the electron-accepting capability of the soluble molecular oxygen (4 × 236 μM at 30°C) exceeded that of the electron-donating capacity of the soluble ferrous ions (≤500 μM), the spectrum of each iron-reduced organism returned to that of the resting air-oxidized organism.

In this study, the same ICAM was exploited to test the hypothesis that different types of redox-active prosthetic groups and electron transfer biomolecules were expressed by microorganisms from each of the six phyla that contain acidophilic members that respire aerobically on iron. This hypothesis was consistent with the observations that (i) *L. ferrooxidans* and *A. ferrooxidans*, which represent the phyla Nitrospirae and Proteobacteria, respectively, produced completely different spectral changes when their intact cells were exposed to iron (Blake and Griff, 2012; Li et al., 2015) and (ii) a number of other different redox-active biomolecules have been implicated in iron-oxidizing acidophiles that are currently assigned to other phyla. We tested this hypothesis by characterizing the cellular absorbance changes that occurred when soluble iron was mixed with cells of two different organisms derived from each of the six phyla that contain members that respire aerobically on iron. A total of six different patterns of iron-dependent absorbance changes were observed: three in the Gram-negative eubacteria; two in the archaea; and one in the Gram-positive eubacteria. We conclude that there are as many as six different sets of prosthetic groups and biomolecules that can accomplish aerobic respiration on soluble iron.

## Materials and Methods

### Cell Culture

*Acidithiobacillus ferrooxidans* ATCC 23270^T^, *L. ferrooxidans* DSM 2705^T^, and *L. ferriphilum* DSM 14647^T^ were cultured autotrophically on soluble ferrous ions at 30°C in the medium described elsewhere (Tuovinen and Kelly, 1973), adjusted to 158 mM FeSO_4_⋅7H_2_O and pH 1.5. *Acidihalobacter ferrooxidans* 14175, *Sulfobacillus thermosulfidooxidans* 9293^T^, *Alicyclobacillus ferrooxydans* 22381^T^, *Acidimicrobium ferrooxidans* 10331^T^, *Ferrimicrobium acidiphilum*, 19497^T^, *Acidiplasma aeolicum* 18409^T^, *Ferroplasma acidiphilum* 12658^T^, *Metallosphaera sedula* 5348^T^, and *Sulfolobus metallicus* 6482^T^ were all obtained from the DSM. Each organism was cultured on 20 mM ferrous sulfate at pH 1.5 using the relevant mixotrophic medium and growth temperature recommended for each microorganism in the DSM media guide. Cells grown to late stationary phase were harvested by centrifugation, washed twice with 0.02 M H_2_SO_4_, and resuspended in sufficient 0.02 M H_2_SO_4_ to achieve a stock suspension of approximately 1 × 10^10^ cells/ml. Each stock suspension was stored at 4°C for no longer than a week while spectroscopic experiments were conducted on aliquots of the cells.

### Quantification of Microorganisms

Absolute numbers of microorganisms were determined by electrical impedance measurements in a Multisizer 4 particle counter (Beckman Coulter, Inc., Brea, CA, USA) fitted with a 30-μm aperture (Blake and Griff, 2012; Li et al., 2015). The instrument was programmed to siphon 50 μl of sample that contained Isoton II as the electrolyte. The current applied across the aperture was 600 μA. Voltage spikes attendant with impedance changes as microorganisms passed through the aperture were monitored with an instrument gain of four.

### Absorbance Measurements with Cell Suspensions

Absorbance measurements on intact cells in suspension were conducted in an OLIS CLARiTY 1000A spectrophotometer (On Line Instrument Systems, Inc., Bogart, GA, USA) as described previously (Blake and Griff, 2012; Li et al., 2015). In a typical experiment, identical 8-ml solutions of 0.02 M sulfuric acid, pH 1.5, were added to both the sample and reference observation cavities of the spectrophotometer. A volume was withdrawn from the sample cavity and replaced with an equal volume of suspended cells. The contents of both observation cavities were maintained at the respective growth temperature of each organism using a model TC-1 Peltier temperature control element from Quantum Northwest (Liberty Lake, WA, USA). After recording a stable baseline, 40 μl of a 200 mM solution of ferrous sulfate, pH 1.5, were added to the sample observation chamber to create a 1.0 mM solution in reduced iron. This concentration of the ferrous ion electron donor was always in excess to the electron accepting capacity of the molecular oxygen electron acceptor in the reaction mixtures, which ranged from 236 to 150 μM in the mixtures at temperatures from 30 to 65°C, respectively. Raw absorbance spectra were subsequently collected at a rate of 6.2/s for several minutes. Iron-dependent absorbance changes in the suspensions of cells were complete in the time that it took to add the electron donor, close the chamber, and initiate the data collection. The resulting absorbance changes were stable for the entire time that raw data were collected. These raw absorbance values were subsequently converted to equivalent absorbance values per cm using Fry’s method (Fry et al., 2010) with analysis software provided by OLIS, Inc.

## Results

### Integrating Cavity Absorption Meter

The principal features of the ICAM employed herein to conduct absorbance measurements on intact cells in turbid suspensions were described earlier (Blake and Griff, 2012; Li et al., 2015). This novel design for a spectrophotometer imposes two consequences. First, the multiple transversals around the interior of the observation cavity means that the incident exciting light experiences a much longer effective path length than it would in an equivalent linear spectrophotometer where the transmitted light experiences only one pass through a filled cuvette. Consequently, absorbance measurements in this ICAM result in a much greater sensitivity than would equivalent measurements in a standard linear spectrophotometer. Second, to the extent that the exciting incident light can be made to be totally diffuse, there are no longer deleterious consequences to absorbance measurements from turbid suspensions that scatter light. If the exciting light is already randomly scattered to a maximum extent, then additional light scattering by the turbid sample will have no immediate consequences on the integrity of the absorbance measurement. Thus it is possible to conduct absorbance measurements *in situ* in whole cells under physiological solution conditions.

### Gram-Negative Eubacteria

The Proteobacteria and the Nitrospirae are the two phyla of Gram-negative eubacteria that contain obligately acidophilic members that respire aerobically on soluble iron (Bonnefoy and Holmes, 2012). These bacteria are distributed among at least four genera in the Proteobacteria: *Acidithiobacillus, Acidiferrobacter, Acidihalobacter* and *Ferrovum*. There are four species recognized within the genus *Acidithiobacillus* that oxidize iron, while there are at least two species within the genus *Acidihalobacter* that also do so. We chose *Ah. ferrooxidans* and *At. ferrooxidans* to represent the Proteobacteria phylum in the *in situ* spectroscopic studies reported herein.

**FIGURE 1:**
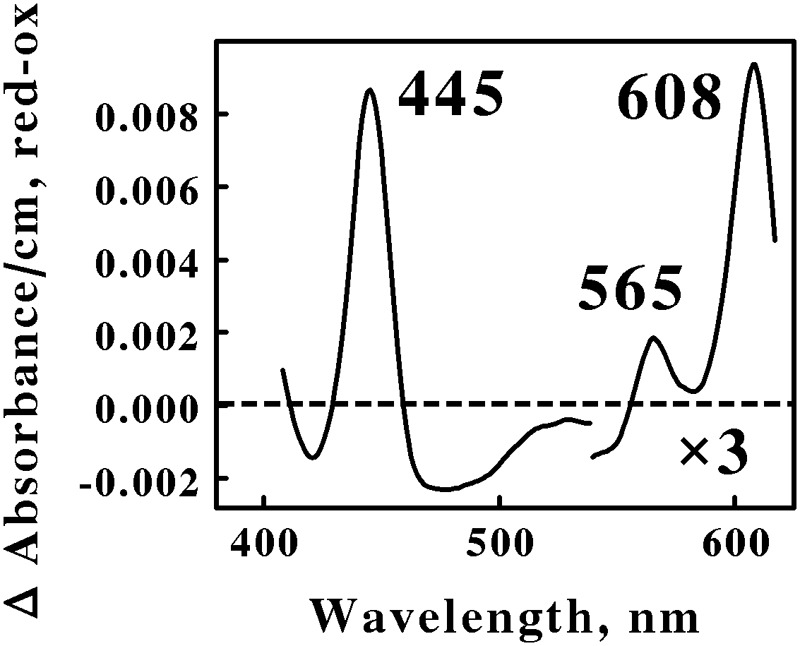
**Reduced minus oxidized difference spectra obtained when excess ferrous sulfate was mixed with intact cells of *Acidihalobacter ferrooxidans*, representing the Proteobacteria.** The number of cells present in 8 ml were 2.7 × 10^9^. The bold numbers identify the wavelengths where each peak exhibited its maximum value. The absorbance values in the α β regions of the spectrum were multiplied by 3 to aid in the clarity of presentation.

**Figure 1** shows the reduced minus oxidized difference spectrum that was observed when intact cells of air-oxidized *Ah. ferrooxidans* were exposed to excess ferrous ions at 35°C and pH 1.5. This difference spectrum was different from that obtained previously with *At. ferrooxidans* (Li et al., 2015) in a number of ways. First, the difference spectrum in **Figure 1** exhibited no evidence of typical reduced cytochrome *c*-dependent absorbance changes around 417 or 550 nm. In contrast, the participation of *c*-type cytochromes in the comparable difference spectrum obtained previously with *At. ferrooxidans* was indicated by prominent peaks at 417, 520, and 551 nm. Second, the reduced peak at 565 nm in **Figure 1** was clearly in the spectral region normally attributed to the reduced peaks of *b*-type cytochromes, a feature that was absent in the spectrum obtained using intact *At. ferrooxidans*. Third, the first α peak encountered in the difference spectrum of *Ah. ferrooxidans* had a reduced peak at 608 nm, some 10 nm red-shifted from the equivalent peak seen in *At. ferrooxidans* at 598 nm. Finally, there was no evidence of a broad trough of negative absorbance in the spectrum in **Figure 1** that one could attribute to the iron-dependent reduction of a rusticyanin-like molecule. In contrast, iron-reduced intact *At. ferrooxidans* exhibited a broad trough of negative absorbance in the difference spectrum from 500 to 650 nm which was consistent with the hypothesis that concentrated amounts of the blue copper protein rusticyanin were reduced by soluble iron.

Spectral properties similar to those shown in **Figure 1** have been reported for terminal oxidases expressed by two other Gram-negative eubacteria: *Thermus thermophilus*, a thermophile that grows at 70°C (Zimmerman et al., 1988); and *Rhodothermus marinus*, a thermohalophilic bacterium (Verissimo et al., 2007). The positions of the reduced α and β peaks varied between these two bacteria from 600 to 613 nm and from 577 to 562 nm, respectively. These absorbance properties were attributed to a cytochrome *ba*_3_ terminal oxidase expressed by these two extremophilic eubacteria. A working hypothesis is that the absorbance properties shown in **Figure 1** represent a cytochrome *ba*_3_-type terminal oxidase that participates in the aerobic iron respiratory chain of the obligately halophilic and acidophilic *Ah. ferrooxidans*. In any case, it is evident that *At*. and *Ah. ferrooxidans* utilize different prominent redox-active prosthetic groups and biomolecules for respiratory electron transfer when they respire aerobically on soluble iron.

*Leptospirillum* is the only genus within the Nitrospirae that is known to contain acidophilic members that respire aerobically on iron. The reduced minus oxidized difference spectra contrasted above were quite different from that observed when intact cells of *L. ferrooxidans* were mixed with excess ferrous ions at 30°C and pH 1.5 (Blake and Griff, 2012).

The difference spectrum obtained with *L. ferrooxidans* had a reduced α peak at the unusual position of 579 nm and represents a unique iron-responsive cytochrome that has not been reported for members of the Proteobacteria (Singer et al., 2008). Spectral changes attributable to blue copper proteins or typical cytochromes *a, b*, or *c* have not been reported in the genus *Leptospirillum* and were not visible in the ICAM spectra reported earlier (Blake and Griff, 2012). A working hypothesis is that members of the Nitrospirae and the Proteobacteria express and utilize different redox-active biomolecules to conduct aerobic respiration on iron.

### Gram-Positive Eubacteria

The Firmicutes and the Actinobacteria are the two phyla of Gram-positive eubacteria that contain obligatory acidophilic members that respire aerobically on soluble iron (Bonnefoy and Holmes, 2012). These acidophilic bacteria are distributed among at least three genera in the Firmicutes: *Sulfobacillus, Alicyclobacillus*, and *Acidibacillus*. *Sulfobacillus* and *Alicyclobacillus* contain at least five and four separate species, respectively, that respire on iron. We chose *S. thermosulfidooxidans* and *Alb. ferrooxydans* to represent the Firmicutes phylum in the *in situ* spectroscopic studies reported herein.

**FIGURE 2:**
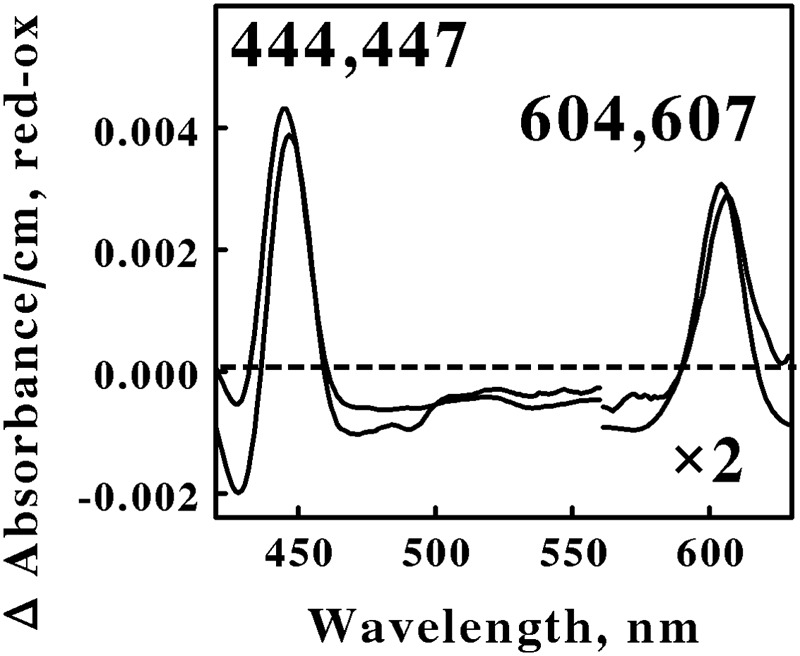
**Reduced minus oxidized difference spectra obtained when excess ferrous sulfate was mixed with intact cells of *Sulfobacillus thermosulfidooxidans* (lower wavelengths) and *Alicyclobacillus ferrooxydans* (higher wavelengths), representing the Firmicutes.** The number of cells present in 8 ml were 6.8 × 10^10^ and 4.4 × 10^10^ for *S. thermosulfidooxidans* and *A. ferrooxydans*, respectively. The absorbance values in the α regions of both spectra were multiplied by 2 to aid in the clarity of presentation.

**Figure 2** shows the reduced minus oxidized difference spectra that were observed when intact cells of *S. thermosulfidooxidans* and *Alb. ferrooxydans* were mixed with excess ferrous ions at pH 1.5 and 50 and 30°C, respectively. Although the two difference spectra differed by several nanometers in both their reduced Soret and α peaks, we judged the two spectra to be sufficiently similar so as to represent the same type of heme prosthetic group embedded in slightly different protein environments. Because the positions of both reduced α peaks were greater than 600 nm, our hypothesis is that the spectra in **Figure 2** represent the respective terminal oxidases in the aerobic iron respiratory chains of these two Firmicutes.

**FIGURE 3:**
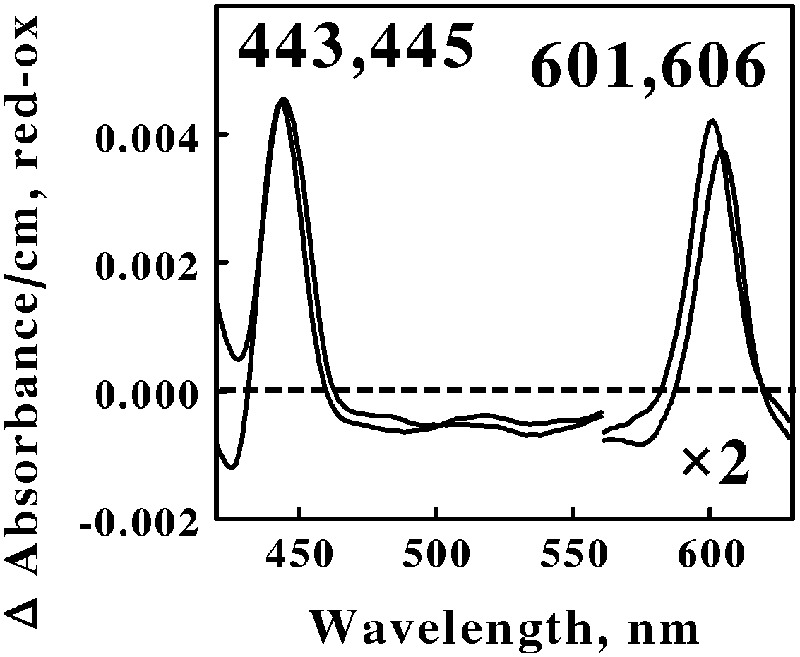
**Reduced minus oxidized difference spectra obtained when excess ferrous sulfate was mixed with intact cells of *Ferrimicrobium acidiphilum* (lower wavelengths) and *Acidimicrobium ferrooxidans* (higher wavelengths), representing the Actinobacteria.** The number of cells present in 8 ml were 3.7 × 10^10^ and 3.1 × 10^10^ for *F. acidiphilum* and *A. ferrooxidans*, respectively. The absorbance values in the α regions of both spectra were multiplied by 2 to aid in the clarity of presentation.

Acidophilic bacteria that respire aerobically on soluble iron are distributed among as least five genera in the phylum Actinobacteria: *Acidimicrobium, Ferrimicrobium, Ferrithrix, Acidithrix*, and perhaps *Acidithiomicrobium*. We chose *Am. ferrooxidans* and *Fm. acidiphilum* to represent the Actinobacteria phylum in the *in situ* spectroscopic studies reported herein. **Figure 3** shows the reduced minus oxidized difference spectra that were observed when intact cells of *Am. ferrooxidans* and *Fm. acidiphilum* were mixed with excess ferrous ions at pH 1.5 and 45° and 32°C, respectively. As was the case with the two spectra shown in **Figure 2**, the two difference spectra shown in **Figure 3** differed by only a few nanometers in both their reduced Soret and α peaks. Once again, we judged the two spectra in **Figure 3** to be sufficiently similar so as to represent the same type of heme prosthetic group embedded in slightly different protein environments.

All four of the difference spectra shown in **Figures 2** and **3** are highly similar. The wavelengths of maximum absorbance of the reduced Sorets and α peaks in all four difference spectra support the hypothesis that each spectrum represents an *a*-type heme that is part of the terminal oxidase in its respective microorganism. The slight differences among the four spectra are presumed to be due to subtle structural differences among the respective globins that bind and function using the same *a*-type porphyrins. Thus Gram-positive eubacteria that respire aerobically on iron do so using basically the same principal redox-active prosthetic group in their respective terminal oxidases, regardless of the phylum or genus into which the bacterium is assigned on the basis of the sequence of its 16S ribosomal RNA. We posit that the Gram-positive eubacteria express a unique type of electron transfer pathway and strategy to accomplish aerobic respiration on soluble iron.

### Archaea

The Crenarchaeota and Euryarchaeota are the two phyla of Archaea that contain obligately acidiphilic members that respire aerobically on soluble iron (Bonnefoy and Holmes, 2012). These Archaea are distributed among two genera in the acidophilic Euryarchaeota: *Acidiplasma* and *Ferroplasma*. *Acidiplasma* and *Ferroplasma* contain at least two and three separate species, respectively, that respire aerobically on soluble iron. We chose *Ap. aeolicum* and *Fp. acidiphilum* to represent the Crenarchaeota phylum in the *in situ* spectroscopic studies reported herein.

**FIGURE 4:**
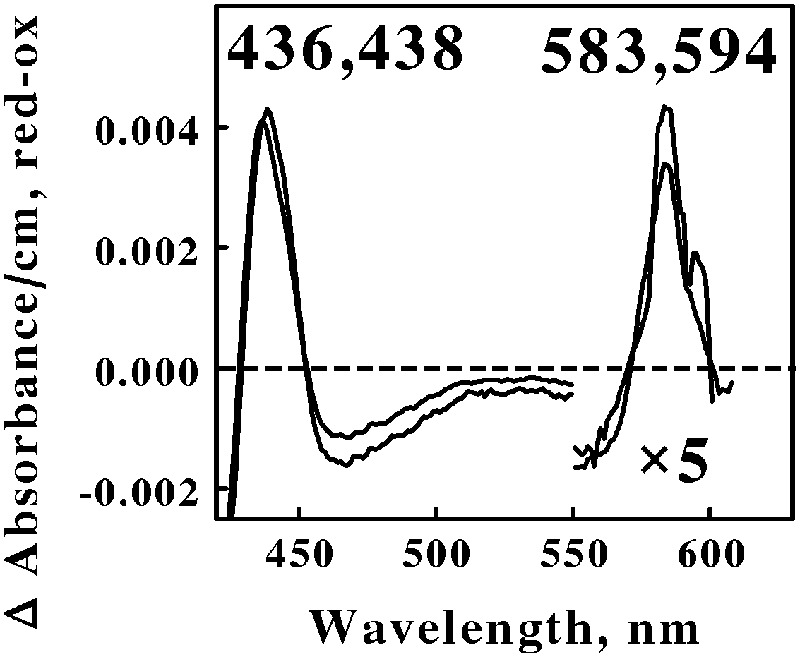
**Reduced minus oxidized difference spectra obtained when excess ferrous sulfate was mixed with intact cells of *Acidiplasma aeolicum* (lower wavelengths) and *Ferroplasma acidiphilum* (higher wavelengths), representing the Euryarchaeota.** The number of cells present in 8 ml were 8.8 × 10^10^ and 9.5 × 10^10^ for *A. aeolicum* and *F. acidiphilum*, respectively. The absorbance values in the α β regions of both spectra were multiplied by 5 to aid in the clarity of presentation.

**Figure 4** shows the reduced minus oxidized difference spectra that were observed when intact cells of *Ap. aeolicum* and *Fp. acidiphilum* were mixed with excess ferrous ions at pH 1.5 and 40° and 35°C, respectively. The absorbance of the reduced α peak observed with *Ap. aeolicum* had a maximum value at 583 nm, but there was also a discernible shoulder at longer wavelengths. The corresponding α peak observed with iron-reduced *Fp. acidiphilum* exhibited two distinct peaks of absorbance, a more intense peak at 583 nm and a less intense peak at 594 nm. Others have reported the existence of an *a*_583_*aa*_3_-type of terminal oxidase in *S. tokodaii*, a member of the phylum Euryarchaeota (Iwasaki et al., 1995; Schafer, 1996; Schafer et al., 1996). The *a*_583_ component in the latter organism is also represented as heme A_S_, an *a*-type heme with a formyl group on ring 1 and a hydroxyethylgeranylgeranyl side chain on ring 2 of the *a*-heme frame (Lubben and Morand, 1994). The accompanying *aa*_3_ component of the terminal oxidase in *S. tokodaii* had a reduced α peak at 603 nm. Because the existence of a reduced α peak at 583 nm is rare, we hypothesize that the terminal oxidases expressed by these two members of the Crenarchaeota represent a novel *a*_583_*aa*_3_-type of terminal oxidase where the *aa*_3_ component differs slightly from that expressed in *S. tokodaii*.

**FIGURE 5:**
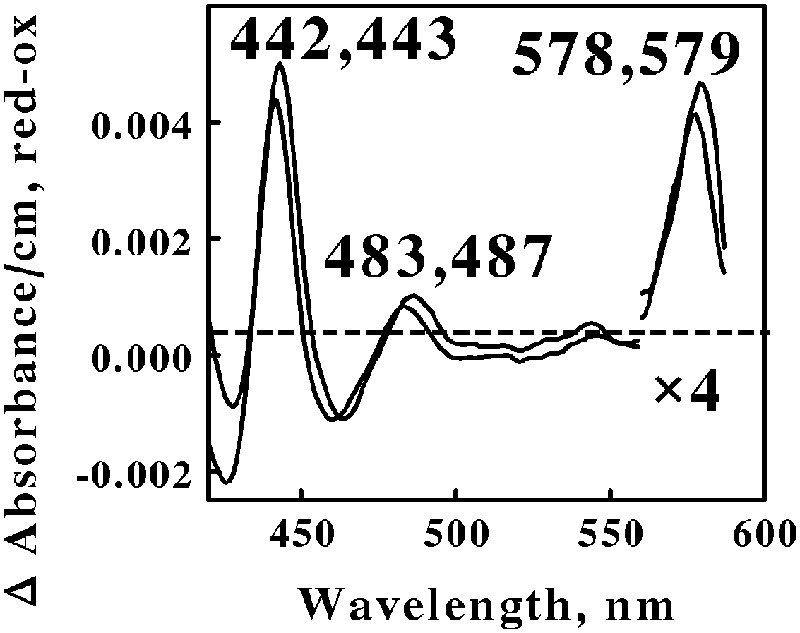
**Reduced minus oxidized difference spectra obtained when excess ferrous sulfate was mixed with intact cells of *Metallosphaera sedula* (lower wavelengths) and *Sulfolobus metallicus* (higher wavelengths), representing the Crenarchaeota.** The number of cells present in 8 ml were 7.3 × 10^10^ and 1.2 × 10^11^ for *M. sedula* and *S. metallicus*, respectively. The absorbance values in the α regions of both spectra were multiplied by 4 to aid in the clarity of presentation.

Acidophilic bacteria that respire aerobically on soluble iron are distributed among four genera in the phylum Euryarchaeota: *Metallosphaera, Sulfolobus, Acidianus*, and *Sulfurococcus*. We chose *M. sedula* and *S. metallicus* to represent the Euryarchaeota phylum in the *in situ* spectroscopic studies reported herein. **Figure 5** shows the reduced minus oxidized difference spectra that were observed when intact cells of each Archaea were mixed with excess ferrous ions at pH 1.5 and 60°C. Although the two difference spectra differed by several nanometers in all three peaks, we judged the two spectra to be sufficiently similar so as to represent the same prosthetic groups embedded in slightly different protein environments.

The first observation was that the reduced Soret and α peaks at around 422/423 and 578/579 nm, respectively, were remarkably similar to those reported for *L. ferrooxidans* (Singer et al., 2008; Blake and Griff, 2012). Thus one can hypothesize that the same heme prosthetic group is expressed and utilized for aerobic respiration on soluble iron by members of both the eubacterial Nitrospirae and the archaeal Euryarchaeota phyla. Interestingly, there is no evidence for genes in *M. sedula* 5348^T^, *M. cuprina* Ar-4, or *S. metallicus* 6482^T^ that encode a protein similar to the cytochrome 579 that is expressed in *L. ferrooxidans* 2705^T^.

The second observation was that no spectral evidence was obtained for the participation of an *a*_583_*aa*_3_-type of terminal oxidase in either of these two representatives of Euryarchaeota, despite its existence in *S. tokodaii* (another member of the Euryarchaeota) and the participation of this latter terminal oxidase during respiration in the Crenarchaeota. The third observation was that a novel spectral species with a reduced peak at around 485 nm was observed when both Euryarchaeota were mixed with an excess of soluble ferrous ions. No evidence for a similar spectral peak was evident from the data reported for *Leptospirillum*. We know of no redox-active prosthetic group that exhibits a reduced peak in the vicinity of 485 nm. We hypothesize that this unexpected spectral intermediate represents a heretofore unknown and uncharacterized prosthetic group that is expressed and exploited by these Euryarchaeota as they respire aerobically on soluble iron.

## Discussion

Our absorbance measurements using a novel integrating observation cell to negate deleterious light-scattering effects in turbid suspensions enabled us to observe those redox-active colored prosthetic groups that were reduced when live cells were exposed to soluble iron under physiological conditions. Any reduced prosthetic group that is observed under these conditions must necessarily play a part in the respiratory electron transfer pathway of that microorganism that conducts electrons from extracellular iron to either intracellular oxygen or onto pyridine nucleotides to generate reducing power within the cell. Further, the actual observance of the reduced form implies that the subsequent oxidation of the prosthetic group in question must be slower than its prior reduction, otherwise the transient reduced form might not achieve a sufficient concentration to be detected using absorbance measurements. This type of situation may account for our inability to detect cytochrome *b* using the ICAM in intact *At. ferrooxidans* and other eubacteria and archaea, where a *b*-type cytochromes have been implicated in the electron transfer pathways required to reconstitute the reducing pool necessary for anabolic processes. We observed a number of different colored prosthetic groups in the observations reported above: *b*- and *c*-type cytochromes; numerous *a*-type cytochromes with reduced peaks from 583 to 608 nm; a blue copper protein, a cytochrome with a reduced peak at 579 nm; and an unknown prosthetic group with a reduced peak at around 485 nm. There are simply too many different prosthetic groups in the cornucopia of colored proteins documented above for all of them to represent different rate-limiting steps in a generic respiratory chain that contains all of these components. It is evident that multiple types of respiratory chains must exist among different microorganisms.

We propose that the data summarized herein are consistent with the hypothesis that at least six different strategies exist in acidophilic microorganisms to conduct aerobic respiration on soluble iron. Each stratagem is characterized by a different set of redox-active prosthetic groups. At least three electron transfer strategies were evident in the Gram-negative eubacteria, which must conduct electrons across a periplasmic space. Still further differences may exist within the four highly related taxa of iron-oxidizing acidithiobacilli, where at least two different pathways for iron oxidation have been proposed to reconcile models that had previously been considered to be conflicting (Amouric et al., 2011). At least two electron transfer strategies were evident in the Archaea, which have only a single plasma membrane to cross. Given the apparent diversity of electron transfer pathways expressed by members in these latter four phyla, it is perhaps surprising that only a single electron transfer strategy may exist in members contained within the two phyla of Gram-positive eubacteria. In any event, the simple picture of a highly conserved universal mechanism for respiratory iron oxidation is clearly inaccurate. It is likely that we do not yet possess a complete inventory of all the redox-active prosthetic groups nor all the microorganisms that participate in respiratory iron oxidation in acidic environments. One should perhaps consider these *in situ* spectroscopic analyses of the most conspicuous components of each respiratory chain as a first step in a more detailed investigation of each unique type of respiratory chain.

The cell wall architectural features of the Gram-negative, Gram-positive and archaea microorganisms are so structurally different that it is difficult to imagine how all three types of organisms could express the identical biomolecules to conduct electrons from the exterior of the cell to their interior to accomplish oxidative phosphorylation. Gram negative eubacteria contain a relatively thin peptidoglycan layer adjacent to their plasma membrane. This is responsible for the cell wall’s inability to retain the crystal violet stain upon decolorization with ethanol-acetic acid during Gram staining. In addition to their thin peptidoglycan layer, the Gram negative bacteria also contain an outer membrane comprised of phospholipids and lipopolysaccharides (Beveridge, 1999). Gram positive eubacteria generally contain a single relatively thick layer of peptidoglycan that comprises a rigid cell wall around the outside of their plasma membrane. Most archaea possess a plasma membrane and an outer cell wall that is assembled from surface-layer proteins, which form a so-called S-layer (Sara and Sleytr, 2000). An S-layer is typically a rigid array of protein molecules that cover the outside of the archaeal cell like chain mail (Engelhardt and Peters, 1998); S-layers have been reported for the *Sulfolobus* and *Metallosphaera* genera (Veith et al., 2009). Notable exceptions to this general structural motif among archaea are that the three known species of *Ferroplasma* exhibit no S-layer and are simply bounded by a mere plasma membrane (Golyshina et al., 2000).

When considering the structural features that might be of greatest relevance to the initial electron transfer reactions that occur between bulk extracellular ferrous ions and cellular electron acceptors, the most immediate differences among these three cell types are the natures of the corresponding periplasmic spaces. The periplasm is the space bordered by the inner and outer membranes in Gram negative bacteria. Strictly speaking, there is no periplasmic space in Gram positive bacteria because there is only one biological membrane, the plasma membrane. However, a region termed the ‘inner wall zone’ has been observed between the plasma membrane and the mature peptidoglycan cell wall (Matias and Beveridge, 2005; Zuber et al., 2006). Similarly, a region termed a ‘quasi-periplasmic space’ has been observed in TEM images between the plasma membrane and the S-layer in archaea (Baumeister and Lembcke, 1992).

Given the significant differences in the outer architectural features of these phylogenetically diverse microorganisms, the hypothesis that was tested in the comparative results presented above is that microorganisms with different types of cell walls will express different electron transfer proteins to respire aerobically on extracellular ferrous ions. Because one might expect different electron transfer proteins to conduct electron transfer reactions at different rates, the next working hypothesis is that different genera of iron-oxidizing bacteria will catalyze the oxidation of iron or the reduction of molecular oxygen at different rates. The test of this hypothesis would be to carefully quantify and compare the kinetic properties of aerobic respiration on iron by each of the intact microorganisms whose *in situ* spectral changes are reported herein. In so doing, one should focus on quantifying both the oxidation of ferrous ions (and/or the appearance of ferric ions) and the consumption of molecular oxygen. In this way, one could achieve a good estimate of the partition of respiratory electrons between oxygen reduction and the reduction of pyridine nucleotides. Such kinetic studies might also reveal any competitive advantages that particular types of respiratory chains might enjoy in terms of catalytic efficiency (turnover number) or affinities for iron or oxygen. Cell wall structural features aside, the evident question is why microorganisms apparently use so many different strategies to conduct aerobic respiration on iron.

## Author Contributions

RB wrote the manuscript and directed the project; he also collected and interpreted data for *Acidithiobacillus ferrooxidans, Alicyclobacillus ferrooxydans*, and both *Leptospirillum ferrooxidans* and *L. ferriphilum*. MA collected and interpreted data for *Ferrimicrobium acidiphilum*. JB collected and interpreted data for *Acidimicrobium ferrooxidans*. TH and BK collected and interpreted data for *Ferroplasma acidiphilum*. KH and BL collected and interpreted data for *Acidiplasma aeolicum*. ML and RP collected and interpreted data for *Sulfobacillus thermosulfidooxidans*. RP also collected and interpreted data for *Metallosphaera sedula* and *Sulfolobus metallicus*.

## Conflict of Interest Statement

The authors declare that the research was conducted in the absence of any commercial or financial relationships that could be construed as a potential conflict of interest.
